# A decade of discovery: bridging science and clinical practice in biomedical research

**DOI:** 10.1016/j.ebiom.2024.105469

**Published:** 2024-11-13

**Authors:** eBioMedicine

Given the complexity of human biological systems, there is often a disconnect between what we conclude from early disease models and how well these assumptions might play out in human clinical trials. At *eBioMedicine*, we prioritise preclinical research with demonstrable relevance to human health and disease, and we place an equally high value on clinical studies that reveal flaws in our understanding of disease mechanisms and target pathways. In this sense, translational research is often a two-way and iterative endeavour. Hypotheses are tested in the laboratory, refined through real-world clinical insights, and then tested again back in the laboratory with the underlying goal of developing effective and targeted therapies. The cover image of our November issue represents the bi-directional synergy that can happen when researchers and clinicians work towards the common goal of improving human health.

Every field of research has its challenges, and advancing towards clinically viable therapies often benefits from a cross-disciplinary approach that can bring a new perspective. There have been numerous examples of collaboration between disciplines leading to substantial translational breakthroughs. Bacteriologists and molecular biologists joined forces to reveal the potential of innate immune responses in bacteria, culminating in the development of CRISPR-Cas9, which has immense promise for treating genetic disorders. Another notable example is the interdisciplinary collaboration between neuroscientists, bioengineers, and computer scientists that led to the creation of brain–machine interfaces that might help to restore movement in people who are paralysed.

To commemorate the tenth anniversary of *eBioMedicine*, and in the spirit of fostering collaborative synergy, we invited several members of our research community to share insights into key challenges, goals, and developments in their fields during the past decade.

Harrer and colleagues discussed the fascinating interface between large-language models and multimodal biological systems. This emerging field of generative biology shows the promise of artificial intelligence (AI) to help make sense of complex biology and to use this knowledge to enable predictive functionality and unprecedented biological engineering. Bai and colleagues highlighted the power of AI tools, such as convolutional neural networks, to analyse large amounts of imaging data with speed and precision, identifying patterns and anomalies that could support the work of radiologists. Ensuring accuracy with diverse and high-quality datasets and maintaining high standards of trust and reliability are crucial challenges when implementing AI in biomedical research.

Schiattarella and colleagues discussed the interconnectedness of cardiovascular and metabolic disease—focusing on heart failure with preserved ejection fraction and metabolic dysfunction-associated steatotic liver disease, which share common risk factors such as obesity and systemic inflammation. In addition to traditional risk factors, the authors note that emerging threats, such as environmental factors (eg, air pollution and microplastics) and climate change, are increasingly recognised as substantial contributors to the exacerbation of the cardiometabolic-disease burden.

Trasande continues on the topic of microplastics, highlighting the serious and numerous health risks posed by the harmful chemicals used to make plastics that have now been linked to a wide range of health concerns, including reproductive conditions, obesity, cardiovascular disease, asthma, autoimmune conditions, and cancers. Trasande notes that major challenges in the field are to develop standardised methods to detect and quantify microplastics and nanoplastics and to develop cost-effective and accessible testing for plastic-related chemicals, such as phthalates, perfluoroalkyl and polyfluoroalkyl substances, and bisphenols. Integrating testing into clinical care also requires health-care staff to be aware of the risks to health and health-care systems to be willing to cover costs. Improved understanding of the mechanisms by which microplastics and plastic-associated chemicals cause disease is also urgently needed—particularly because of their ubiquity in the environment and detection in various human tissues.

Fossil fuels are an integral component of plastic production, which provides a direct link between plastics and one of the most existential threats known to human health—climate change. Schlader and colleagues discuss one aspect of this threat, extreme heat, and its potential to negatively affect the health of vulnerable populations, such as people who work outdoors. As extreme heat becomes more intense and more common, the authors argue for a need to not only improve access and regulatory mandates around cooling resources in the workplace, but also to identify and develop non-cooling-focused treatments that could target the molecular effects of heat stress and improve thermoregulatory capacity.

We invite you to peruse the full collection of tenth anniversary vignettes from our research community in this issue. Similar articles highlighting the challenges and aspirations from other research fields are planned for future issues.

As 2024 comes to an end, we proudly celebrate 10 years of publishing work from a community of investigators spanning the entire spectrum of human-health research. We are deeply grateful to our authors for entrusting us with the peer-review of their work. Our successes during the past decade would also not be possible without the invaluable contributions of our advisors and reviewers. Their insights and guidance have been instrumental in shaping the direction of the journal, empowering us to make well informed editorial decisions. We also extend our most heartfelt gratitude to our loyal readership for their curiosity and engagement during the past decade. To our remarkable *eBioMedicine* community: thank you. We can't wait to see where the next 10 years will take us.

